# Neuralgia

**Published:** 1896-03

**Authors:** 


### Neuralgia.

I was called to a case of "neuralgia of the fifth pair," which had been giving a patient much trouble for months, and the resident physician had been in the habit of using hypodermic injections of morphia, which would end an attack of this stubborn pain, says a writer in the *Medical Brief.* Unfortunately for me, I discovered my hypodermic tablets of morphia were *non est.*

I called for hot water and got my syringe heated, and used the syringe full of simply hot water, and in five minutes my case was at ease.

What cured the case, heat or water? I have often repeated it since, and it has at all times given me success. What is the therapeutic action, is it heat or water?

